# A pilot longitudinal spectral CT study of fructose-related bone microstructure in rats: exploring technical advantages

**DOI:** 10.3389/fphys.2026.1857528

**Published:** 2026-06-18

**Authors:** Xue Lv, Yuqin Hong, Qiao Liu, Na Yu

**Affiliations:** Department of Radiology, The Third Affiliated Hospital of Chongqing Medical University(FangDa Hospital), Chongqing, China

**Keywords:** bone microstructure, fructose, *in vivo* imaging, rat, spectral CT

## Abstract

**Purpose:**

Assessing the unique advantages of spectral CT as a real-time, dynamic, and *in vivo* monitoring technique for investigating fructose intake-related bone microstructural changes in rats.

**Methods:**

Eighteen 3-week-old male Sprague-Dawley rats were randomized into experimental (standard chow + 25% fructose water, n=9) and control groups (standard chow + sterile water, n=9) for 8 weeks. Spectral CT scans were performed at weeks 0, 4, and 8. Calcium (Water) [Ca(Water)], Phosphorus (Water) [P(Water)], and Hydroxyapatite (Water) [HAP(Water)] values of the left femur were measured, and their rates of change were calculated. After 8 weeks, left femurs were harvested for distal femur histology. HE staining assessed trabecular parameters: bone volume fraction (BV/TV), trabecular thickness (Tb.Th), trabecular separation (Tb.Sp), and trabecular number (Tb.N). Inter-group comparisons of trabecular parameters, intra- and inter-group comparisons of spectral parameters and their change rates, and correlation analyses between trabecular and spectral parameters were conducted.

**Results:**

At week 8, the experimental group showed significantly higher BV/TV, Tb.Th, and Tb.N, but no difference in Tb.Sp versus controls. Spectral CT parameters were significantly elevated in the experimental group only at week 8. In both groups, parameters at weeks 4 and 8 exceeded baseline, and week 8 values exceeded week 4 values. The rates of change from 4 to 8 weeks (rate of change_4-8_) were significantly lower than the rates of change from 0 to 4 weeks (rate of change_0-4_) in both groups. No inter-group differences were found for rate of change_0-4_, but the experimental group had significantly higher rate of change_4-8_ and the rates of change from 0 to 8 weeks (rate of change_0-8_). At week 8, BV/TV, Tb.Th, and Tb.N were positively correlated with all three spectral CT parameters, with BV/TV showing strong correlations (r = 0.893–0.901, P < 0.001).

**Conclusion:**

Spectral CT, as a real-time, dynamic, and *in vivo* imaging tool, overcomes the limitations of traditional ex vivo studies by providing a novel, non-invasive quantitative research strategy for longitudinally tracking the effects of fructose intake on bone microstructure in rats. Furthermore, it successfully monitored the dynamic process of fructose-promoted bone mineralization over a short-term period.

## Introduction

High fructose intake has been shown to induce disorders of glucose and lipid metabolism, insulin resistance, and oxidative stress (Wang et al., 2015; Bagi et al., 2018; Veličković et al., 2019; Wang et al., 2021), which may indirectly disrupt the dynamic balance between bone formation and resorption, leading to alterations in bone density and microarchitecture (Pereira et al., 2019; Gao et al., 2022). However, the direct mechanisms through which fructose affects bone mineral dynamics remain controversial, necessitating further extensive animal studies for clarification.

Micro-computed tomography (μCT) is currently the gold standard for three-dimensional analysis of bone microstructure. However, its high radiation dose and complex analysis workflow render it unsuitable for longitudinal *in vivo* studies involving the same animal, as it fails to provide temporally dynamic information on the evolution of diseases or interventions (Kim et al., 2021; Li et al., 2023; Deyhle et al., 2025).In recent years, spectral CT has offered a novel perspective in bone microstructural research by leveraging its technical advantages in material decomposition and multi-parameter quantification (Sun et al., 2015; Eibschutz et al., 2024). Nevertheless, the potential of spectral CT in studying fructose-induced abnormal bone microarchitecture in animal models has not been fully explored.

Therefore, this study employs male Sprague-Dawley rats to quantitatively analyze changes in bone microstructure following high-fructose water intake using spectral CT. The aim is to evaluate the technical advantages of spectral CT in research on fructose-related bone microstructural changes in rats and to provide a novel non-invasive imaging strategy for the longitudinal monitoring and assessment of bone microstructure in live animal models.

## Methods

### Animal grouping and interventions

All animal experiments have been verified in accordance with the *Guideline Checklist for Publishing Research Papers on Animal Experimentation and Comparative Medicine in China (2024 Edition)* (Editorial Board of Laboratory Animal and Comparative Medicine, 2024) and were approved by the Animal Experiment Ethics Committee of Chongqing Medical University. Eighteen 3-week-old male Sprague-Dawley rats were purchased from the Experimental Animal Center of Chongqing Medical University (Animal Production License No.: SCXK (Yu) 2022-0010). The rats were housed in sterile cages under controlled conditions: constant temperature (21°C), 50–60% humidity, and a 12-hour light/dark cycle, with free access to food and water. Based on body weight, they were randomly assigned to either an experimental group (standard rat chow + 25% fructose water, n = 9) or a control group (standard rat chow + sterile water, n = 9). The intervention lasted for 8 weeks.

### Imaging protocol

Eighteen rats were scanned under anesthesia at weeks 0, 4, and 8 using a multi-slice spiral CT scanner (Revolution CT, GE Healthcare) in gemstone spectral imaging (GSI) mode. Prior to CT imaging, rats were anesthetized with an intraperitoneal injection of sodium pentobarbital (50 mg/kg). Upon confirmation of the loss of corneal reflex and the absence of a withdrawal response to a hind paw pinch, CT scanning was performed. During scanning, the limbs were extended and fixed in position, with each animal labeled according to group and scanning time. The scanning parameters were set as follows: tube voltage: rapid kVp switching from 80 to 140 kV, tube current: 190 mA, detector width:40 mm, helical pitch: 0.516:1, reconstruction slice thickness and interval: 0.625 mm.

### Image processing

**Figure 1:**
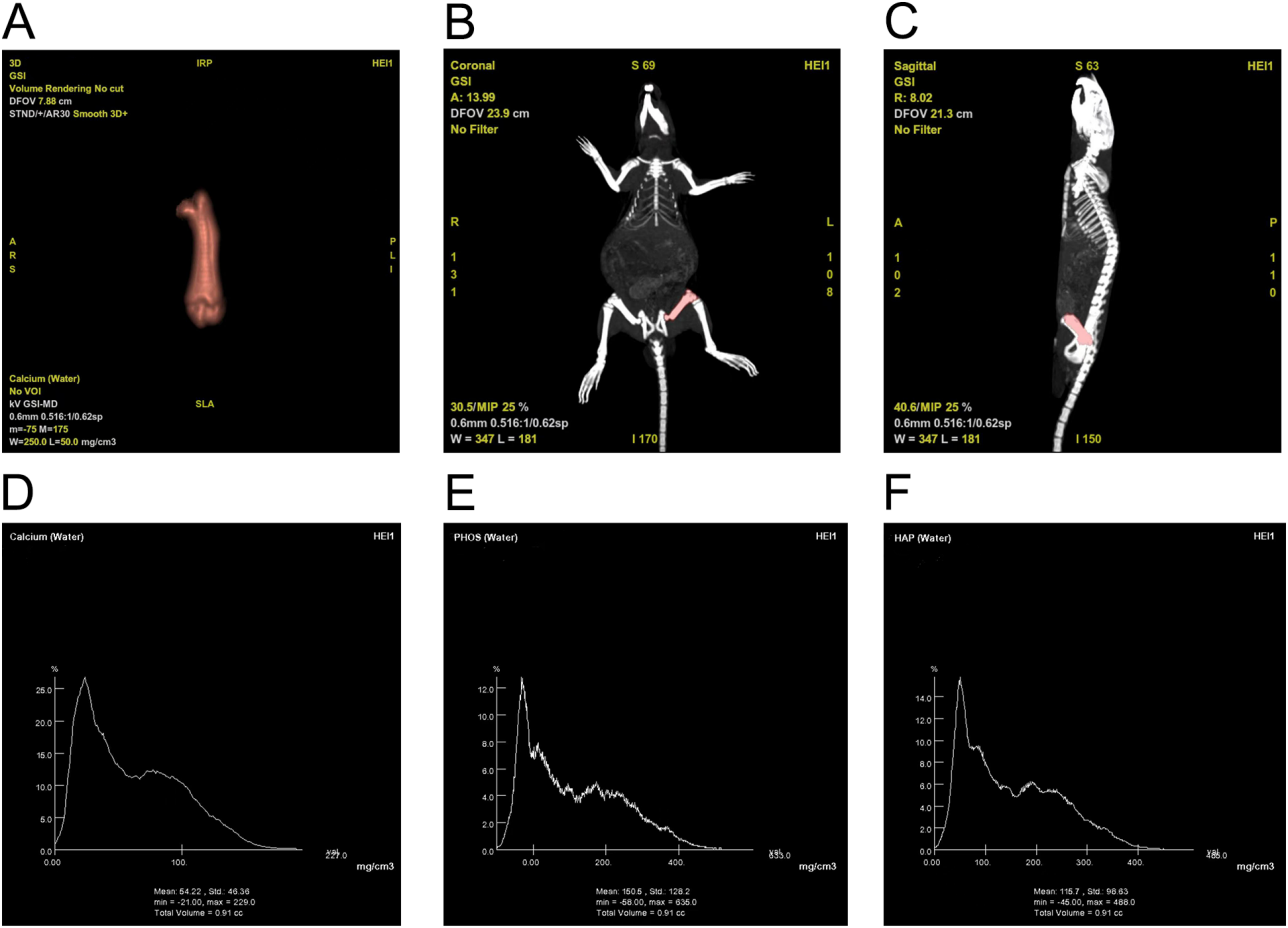
Measurement of spectral CT quantitative parameters. **(A–C)** The left femur of the rat was segmented on spectral CT images to generate VR reconstructions. **(D–F)** Density plots of Ca(Water), P(Water) and HAP(Water), with mean values of 54.22 mg/cm³, 150.5 mg/cm³, and 115.7 mg/cm³, respectively.

Image processing was performed on a workstation (AW 4.7, GE Healthcare). Using a software (GSI Viewer), Volume-rendered (VR) images of the left femurs were reconstructed. The measurements of Ca(Water), P(Water), and HAP(Water) were simultaneously derived using the GSI material decomposition algorithm (**Figure 1**). All density values were independently measured by two radiologists (NY, 20 years; XL, 8 years of experience in CT interpretation), with each observer performing three repeated measurements; the mean values were used for analysis. To assess the consistency between the two radiologists, the intraclass correlation coefficient (ICC) was calculated for each spectral CT parameter. The analysis was performed using Python (pingouin library). The rates of change from 0 to 4 weeks (rate of change_0-4_), from 4 to 8 weeks (rate of change_4-8_), and from 0 to 8 weeks (rate of change_0-8_) for density values were calculated.

### Bone histology

Following the CT scan at week 8, the rats were administered an overdose of sodium pentobarbital (150 mg/kg) via intraperitoneal injection. Death was confirmed after the complete cessation of respiration and heartbeat, which was corroborated by observing pupil dilation, loss of light reflex, and the absence of any physical response to a toe pinch. The left femur was then dissected and harvested.Each specimen was fixed in 4% paraformaldehyde for 24h and then decalcified in 20% ethylenediaminetetraacetic acid (EDTA) until the femurs were completely decalcified.they were subsequently embedded in paraffin and sectioned by the standard histological procedure. Serial sections(3μm) of the distal femurs were prepared and stained with hematoxylin-eosin (H&E).Whole-slide images were acquired using a scanner (Pannoramic MIDI II, 3dhistech, Hungary)and analyzed with a software(Image-Pro Plus 6.0).

### Statistical analysis

Statistical analyses were performed using Python 3.6.8 with relevant scientific computing libraries (**Supplementary Table 1**). Repeated-measures ANOVA (RM-ANOVA) was conducted for each spectral CT parameter, with Time as within-subject factor and Group as between-subject factor, examining main effects and Time × Group interaction. For between-group comparisons at each time point, Bonferroni correction was applied. For within-group pairwise comparisons, Bonferroni correction was applied separately per group and parameter. Rates of change were compared between groups using independent t-test or Mann-Whitney U test, followed by FDR (Benjamini-Hochberg) correction across 9 comparisons. Normality was assessed by Shapiro-Wilk test. Normally distributed variables are presented as mean ± SD, otherwise as median (IQR). A two-sided p < 0.05 was considered statistically significant after correction where applicable. Post-hoc power was calculated based on observed Cohen’s d (threshold ≥ 0.80). Spearman correlation was performed between spectral CT parameters and bone microstructure measurements at week 8.

## Results

### Longitudinal changes in spectral CT parameters

**Figure 2:**
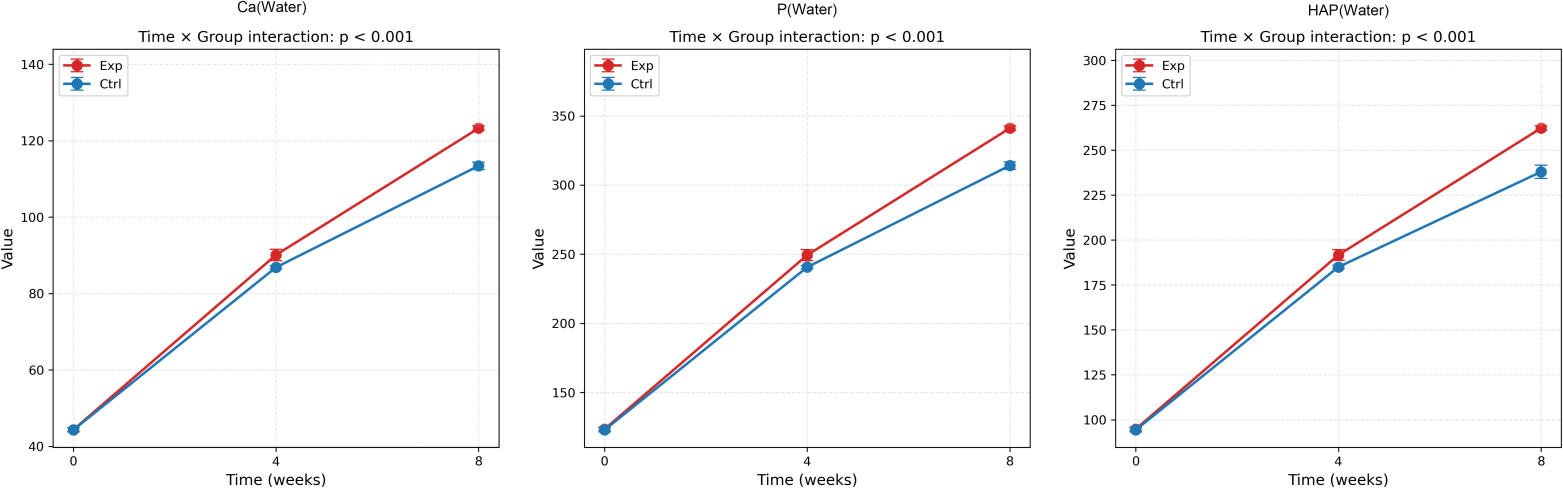
Time-dependent effects of fructose intervention on bone mineral content as assessed by three spectral CT parameters.

RM-ANOVA showed significant main effects of time, main effects of group, and time × group interaction effects for all three spectral CT parameters (p < 0.001), indicating that the effect of fructose intervention on bone mineral content changes over time (**Table 1**; **Figure 2**).

**Table 1:** Repeated-measures ANOVA results.

Parameter	Source	SS	df	MS	F	*p*
Ca (Water)	Group	258.64	1	258.64	23.97	<0.001
	Time	49928.32	2	24964.16	5388.66	<0.001
	Group×Time	225.76	2	112.88	24.37	<0.001
P (Water)	Group	1971.70	1	1971.70	24.04	<0.001
	Time	380539.79	2	190269.90	5833.73	<0.001
	Group×Time	1647.14	2	823.57	25.25	<0.001
HAP (Water)	Group	1483.34	1	1483.34	23.87	<0.001
	Time	220784.94	2	110392.47	3382.18	<0.001
	Group×Time	1377.91	2	688.95	21.11	<0.001

### Comparisons of parameters between groups

Post-hoc power analysis confirmed that the sample size was sufficient to detect these differences (**Supplementary Table 2**). The inter-observer agreement for all spectral parameters was excellent (ICC > 0.90) (**Supplementary Table 3**).

**Figure 3:**
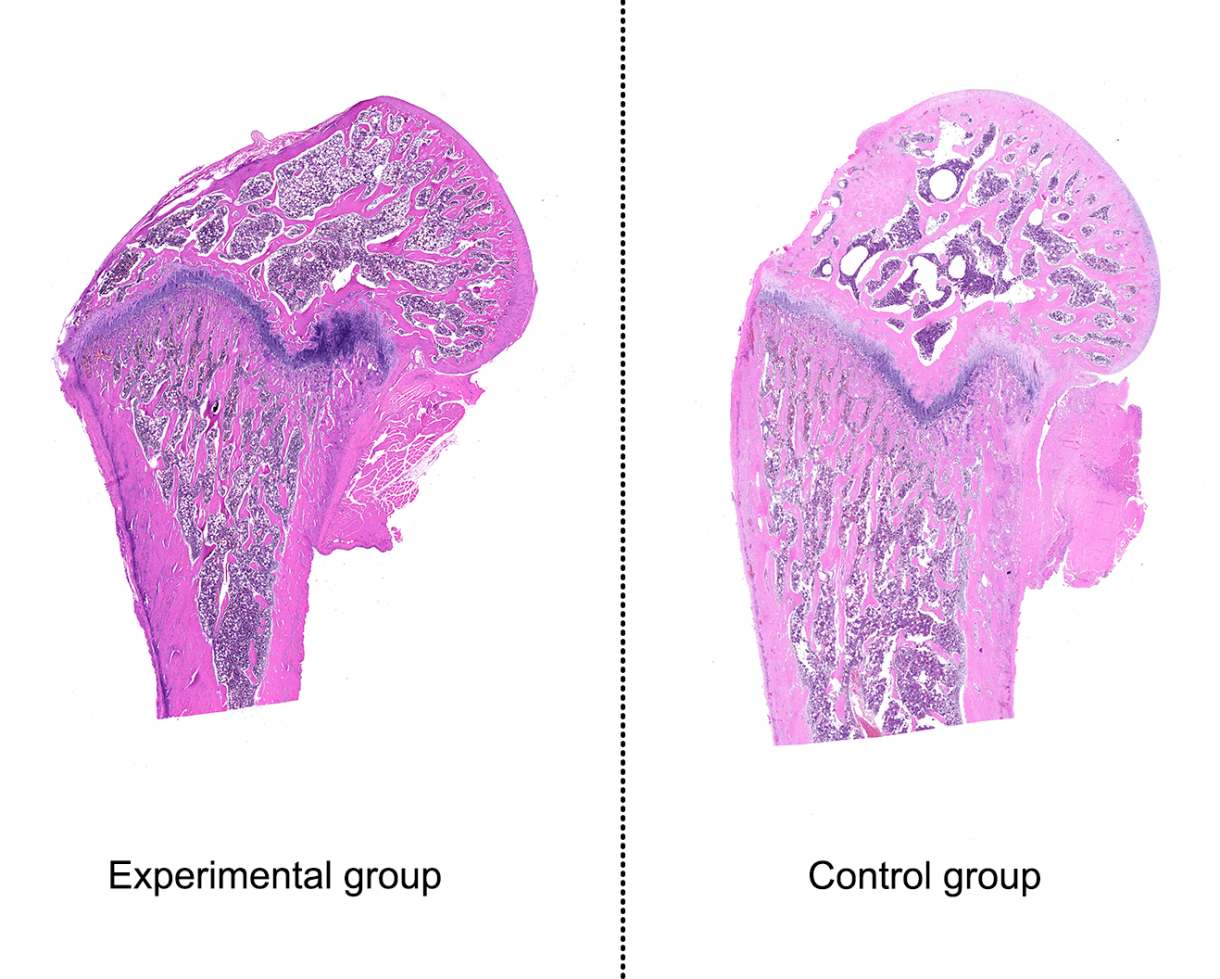
The histological images of the distal femoral bone tissue of the experimental group and the control group at the 8th week.

Bonferroni-corrected between-group comparisons showed that at week 8, significant differences in bone volume fraction (BV/TV), trabecular thickness (Tb.Th), and trabecular number (Tb.N) were observed between the two groups. In contrast, no significant intergroup difference was found in trabecular separation (Tb.Sp) (**Figure 3**). At weeks 0 and 4, there were no significant differences between the experimental group and the control group in all three parameters (**Supplementary Table 4**). However, at week 8, all these parameters were significantly higher in the experimental group than in the control group (**Table 2**).

**Table 2:** Between-group comparisons of parameters at week 8 (Bonferroni-corrected).

Parameter	Experimental group	Control group	*p*_corr
BV/TV (%)	0.38 ± 0.03	0.30 ± 0.02	<0.001*
Tb.Th (μm)	154.93 (109.17,163.52)	76.02 (68.93,88.82)	0.006*
Tb.Sp (μm)	252.74 (186.99,292.29)	184.86 (175.34,195.43)	0.216
Tb.N (mm-1)	7.22 ± 0.78	6.16 ± 0.64	0.006*
Ca (Water) (mg/cm3)	123.28 ± 1.91	113.42 ± 2.96	<0.001*
P (Water) (mg/cm3)	341.09 ± 5.28	314.11 ± 8.13	<0.001*
HAP (Water) (mg/cm3)	262.26 ± 3.98	237.94 ± 11.06	<0.001*

*Statistically significant difference.

### Spectral CT parameters at 0, 4, and 8 weeks

Compared with baseline values (week 0), both groups exhibited significant increases in the values of Ca (Water), P (Water), and HAP (Water) at weeks 4 and 8. Furthermore, compared with those at week 4, all three spectral CT quantitative parameters in both groups were significantly elevated at week 8 (**Supplementary Table 5**).

### Between-group comparisons of the rates of change

**Figure 4:**
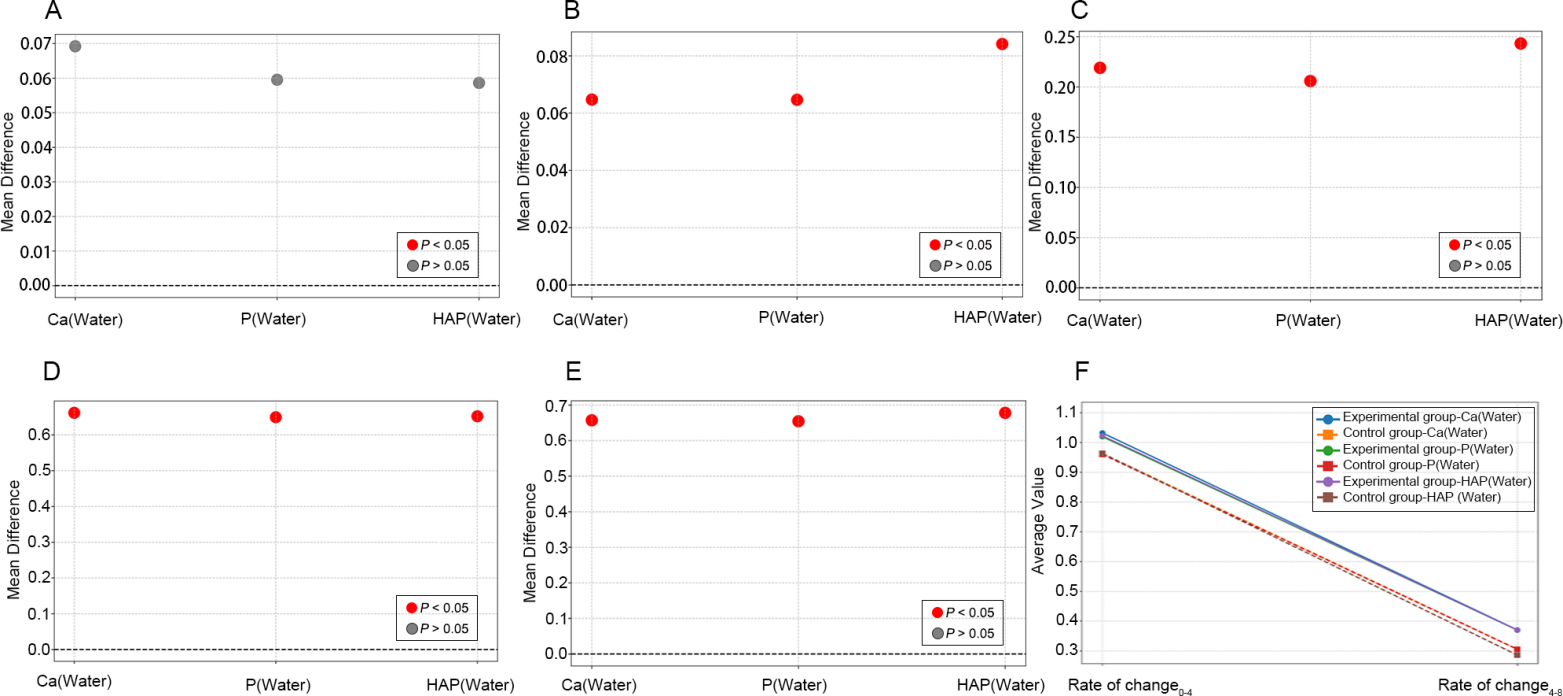
Inter-group and intra-group comparisons of the rates of change for spectral CT parameters. **(A–C)** Inter-group comparisons of the rate of change_0-4_, rate of change_4-8_, and rate of change_0-8_. (**D, E)** Intra-group comparisons of the rate of change_0-4_ and rate of change_4-8_. **(F)** Trend chart illustrating the variation in the rates of change.

Between-group comparisons (FDR-corrected) revealed no significant differences in the rate of change_0-4_ between the two groups (p_FDR > 0.05). However, the experimental group exhibited significantly higher rates of change_4-8_ and rates of change_0-8_ than the control group (p_FDR < 0.01) (**Table 3**; **Figure 4**).

**Table 3:** Between-group comparisons of the rates of change (FDR-corrected).

Rates of change	Experimental group	Control group	*P*-RDF
Rate of change_0-4_			
Ca (Water)	1.03 ± 0.08	1.00 (0.92,1.03)	0.289
P (Water)	1.02 ± 0.08	1.00 (0.91,1.02)	0.289
HAP (Water)	1.02 ± 0.08	1.00 (0.91,1.02)	0.251
Rate of change_4-8_			
Ca (Water)	0.37 ± 0.05	0.31 ± 0.02	0.004*
P (Water)	0.37 ± 0.05	0.31 ± 0.02	0.003*
HAP (Water)	0.37 ± 0.05	0.31 (0.26, 0.32)	0.016*
Rate of change_0-8_			
Ca (Water)	1.78 ± 0.10	1.56 ± 0.13	0.003*
P (Water)	1.76 ± 0.09	1.56 ± 0.13	0.003*
HAP (Water)	1.77 ± 0.10	1.53 ± 0.16	0.004*

*Statistically significant difference.

### Intra-group comparisons of rates of change

In both the experimental and control groups, the rates of change_4-8_ for Ca (Water), P (Water), and HAP (Water) were significantly lower than those of rates of change_0-4_ (**Supplementary Table 6**; **Figure 4**).

### Correlation analysis

At week 8, correlation analysis between spectral CT parameters and bone microstructural measurements revealed that BV/TV, Tb.Th, and Tb.N were all positively correlated with the three spectral CT parameters. Among these, BV/TV demonstrated strong positive correlations with Ca (Water) (r = 0.898, P < 0.001), P (Water) (r = 0.901, P < 0.001), and HAP (Water) (r = 0.893, P < 0.001) (**Table 4**).

**Table 4:** Correlation analysis between spectral CT parameters and bone microstructure measurements.

Trabecular parameter	Ca (water)		P (water)		HAP (water)	
	r	*P*	r	*P*	r	*P*
BV/TV	0.898	<0.001*	0.901	<0.001*	0.893	<0.001*
Tb.Th	0.646	0.004*	0.639	0.004*	0.645	0.004*
Tb.Sp	0.236	0.347	0.216	0.390	0.232	0.354
Tb.N	0.570	0.013*	0.562	0.015*	0.552	0.018*

*Statistically significant difference.

## Discussion

Numerous studies have investigated the effects of fructose on bone microstructure in animals, yet the findings remain inconsistent. One study reported that, compared with glucose-fed rats, fructose-fed rats exhibited greater BV/TV and Tb.Th in the distal femoral trabecular region, along with a significantly higher maximum bending load in the three-point bending test (Bass et al., 2013), which is consistent with our results. However, it is difficult to directly compare our results due to the absence of a standard diet control group in their study. Tian et al. (2018) observed that bone mass in high-fructose-diet (HFRD) mice reached its peak after 16 weeks of feeding compared to the control group, followed by a subsequent decline. This suggests that short-term high-fructose feeding may exert a positive effect on bone mass, whereas long-term intake leads to adverse outcomes. A consistent trend was also reported by Li et al (Li et al., 2023), which may be attributed to the combined effects of body mass, fat mass, bone formation and resorption, as well as proinflammatory factors and the bone marrow microenvironment (Milne and Nielsen, 2000; Felice et al., 2014; Jatkar et al., 2017). The results from Tsanzi et al. (2008) indicated that although the relative femoral weight of rats fed fructose-containing beverages was lower than that of rats provided deionized distilled water (ddH_2_O), no significant differences were observed in femoral morphometry. In contrast, Yarrow et al. (2016) reported that high-fructose diets adversely affected bone in young male Sprague-Dawley rats, but when added to a high-fat diet, the combination did not worsen bone loss. The discrepancies among these findings may be attributed to factors such as fructose dosage,fructose concentration, duration of feeding, and the age of the animals (Tian and Yu, 2017; Chen and Jiang, 2025). To advance our understanding of the impact of fructose on bone microstructure and its underlying mechanisms, the development and application of quantitative measurement methodologies are essential to support extensive animal studies.

μCT is currently the gold standard for three-dimensional bone microstructure analysis (Kim et al., 2021; Li et al., 2023). However, acquiring images at the highest resolution typically requires ex vivo isolation of bone specimens, which precludes longitudinal studies or continuous monitoring within the same animal. Additionally, the procedure involves high radiation exposure (Deyhle et al., 2025). Moreover, μCT instruments themselves, along with their maintenance, are considerably expensive. The process also demands substantial computational power and specialized image analysis software, requiring operators to possess a high level of technical expertise. On the other hand, bone histomorphometry remains indispensable as it offers crucial cellular-level parameters, bridging the gap between two-dimensional and three-dimensional assessments of bone specimens (Li et al., 2023). Nevertheless, the process of preparing bone specimens requires complex procedures and demands a high level of expertise from professionals.

Spectral CT, distinct from conventional μCT, utilizes rapid high- and low-kVp switching technology to generate various monoenergetic images (Ma et al., 2018; Sandvold et al., 2024). Recent studies have demonstrated that virtual monoenergetic images (VMIs) acquired from spectral CT exhibit high accuracy and robustness in quantifying BV/TV (Wili et al., 2025). Furthermore, by leveraging the energy-dependent attenuation characteristics of materials, spectral CT can differentiate and quantify material composition through a process known as material decomposition. This technique decomposes complex substances into combinations of two or three known “base materials” and calculates their respective concentrations (McCollough et al., 2015; Fernández-Pérez et al., 2022; Schilham et al., 2025). Spectral CT’s material decomposition technology shows great potential for quantifying bone microstructure in animal models. It effectively distinguishes bone minerals (e.g., hydroxyapatite) from surrounding tissues, enabling accurate mineral quantification while reducing density overlap and beam hardening artifacts (Greffier et al., 2023). In the present study, spectral CT parameters were measured across the entire femur of rats, providing a more comprehensive and accurate assessment compared to conventional region-of-interest (ROI) placement approaches (Lv et al., 2023; Revel et al., 2004; Zhong et al., 2023).

More than 90% of the inorganic components in bone exist in the form of hydroxyapatite crystals [chemical formula: Ca_10_(PO_4_)_6_(OH)_2_], with calcium (Ca) and phosphorus (P) serving as their fundamental constituent elements (Buckwalter and Cooper, 1987). Therefore, this study selected three base material pairs: Ca (Water), P (Water), and HAP (Water). The results revealed that at week 8, fructose administration led to a significant increase in Ca (Water), P (Water), and HAP (Water) values in the experimental group compared to the control group. Longitudinal observation showed that all three parameters increased significantly over the feeding period in both groups. This reflects an increase in bone mineral content during the early growth of young male rats, suggesting a positive effect of fructose on bone development. Due to the limited sample size, in order to control for individual differences, we calculated the rate of change for three spectral parameters to conduct comparisons between groups and within groups. The results indicated that the rate of change_4-8_ were lower than rate of change_0-4_ in both the experimental and control groups, indicating that fructose did not alter the natural decline in bone growth rate over time. However, the rates of change_4-8_ and the rates of change_0-8_ of Ca (Water), P (Water), and HAP (Water) were significantly higher in the experimental group than in the control group, indicating that fructose promoted the rate of bone mineral deposition to a certain extent. In summary, rats provided with fructose-containing water for eight weeks exhibited superior bone microstructure. These findings are inconsistent with certain previous studies (Milne and Nielsen, 2000; Tsanzi et al., 2008; Felice et al., 2014; Yarrow et al., 2016; Jatkar et al., 2017; Tian et al., 2018; Li et al., 2023), which may be attributed to differences in the growth stage of the animals and varying skeletal sensitivity to fructose (Minematsu et al., 2018). Additionally, since our study lasted only eight weeks, potential adverse effects of fructose might become apparent over a longer duration (Li et al., 2023; Deyhle et al., 2025).

In terms of correlation analysis, the spectral CT parameters demonstrated certain associations with trabecular bone parameters at week 8. Specifically, Ca (Water), P (Water), and HAP (Water) all showed significant positive correlations with BV/TV, Tb.Th and Tb.N, suggesting that these spectral CT parameters may reflect changes in bone microstructure to some extent. However, no statistically significant correlations were observed with Tb.Sp. This lack of significance may be attributed to bias resulting from the limited sample size.

This study has several limitations. First, the relatively small sample size may introduce some bias into the results. Additionally, no *a priori* power analysis was conducted due to the lack of preliminary data on spectral CT parameters in this animal model. Second, the reconstruction slice thickness of 0.625 mm may introduce partial volume effects in small-animal trabecular imaging, potentially leading to some degree of overestimation or underestimation of absolute mineral density values. However, this limitation likely affected both groups equally, and thus the between-group comparisons and relative change rates remain valid. Third, EDTA decalcification may cause tissue shrinkage, which could affect the absolute values of histomorphometric parameters and introduce bias into the histology-imaging correlation analysis. Fourth, the 8-week observation period covers only the early growth phase of young male rats. As reported by Tian et al. (2018), longer-term fructose intake (≥16 weeks) may shift from beneficial to detrimental effects on bone. Therefore, our conclusions should be limited to short-term effects, and extended observation periods are needed in future studies. Fifth, the current analysis lacks serum biochemical markers of bone metabolism, as well as body weight trajectories and inflammatory biomarkers. This limits our ability to directly support the speculative interpretation that short-term fructose-induced bone mineralization is mediated by body mass gain and inflammatory factors. Future studies will incorporate serum markers, longitudinal body weight monitoring, and inflammatory marker assessment to further corroborate the imaging findings and provide functional evidence of bone formation and resorption, as well as to validate the underlying mechanisms. Furthermore, while the present study focused on conventional morphometric indices, future studies may consider applying radiomic texture analysis (e.g., GLCM- or GLSZM-based features) to extract higher-order information from spectral CT images, which could potentially provide more sensitive characterization of trabecular heterogeneity.

## Conclusion

Spectral CT, with its capability for material decomposition and multiparametric quantification, offers significant technical advantages for the study of bone microstructure. It has successfully expanded the research paradigm from static ex vivo analysis to dynamic *in vivo* tracking, enabling real-time, longitudinal observations of bone metabolic processes within the same animal model. This study is the first to apply this technology to dynamically reveal that high fructose intake can accelerate bone mineralization in rats over a short-term period. As a powerful *in vivo* research platform, spectral CT opens up new possibilities for future in-depth investigations into the complex and dynamic effects of nutrition, medication, or diseases on the skeletal system.

## Data Availability

The raw data supporting the conclusions of this article will be made available by the authors, without undue reservation.
