# Supplementary material for: A pilot longitudinal spectral CT study of fructose-related bone microstructure in rats: exploring technical advantages
Source: Front Physiol. 2026 Jun 18;17:1857528. doi: 10.3389/fphys.2026.1857528 (PMC13322858; doi:10.3389/fphys.2026.1857528)
Supplement: Supplementary file 1 [file Supplementaryfile1.docx]

**Supplementary Table S1.** Summary of Python libraries used in the statistical analyses

| Library | Purpose |
| --- | --- |
| pandas | Data management (reading, cleaning, transforming) |
| numpy | Numerical computations (means, standard deviations, arrays) |
| matplotlib | Plotting (line plots, boxplots, scatter plots, heatmaps) |
| seaborn | Enhanced statistical graphics (boxplots, heatmaps, regression plots) |
| scipy | Statistical tests (t-test, Mann-Whitney U, Wilcoxon, Shapiro-Wilk, Levene, Spearman correlation) |
| statsmodels | Multiple comparison correction (Bonferroni, FDR), post-hoc power analysis |
| fpdf | PDF report generation |
| os | File path operations |
| re | Regular expression |
| warnings | Warning control |

**Supplementary Table S2.** Post-hoc power analysis for spectral CT parameters at week 8

| Spectral CT Parameter | Observed Effect Size (Cohen's d) | Achieved Power | Sample Size (n per group) | Alpha |
| --- | --- | --- | --- | --- |
| Ca(Water) | 3.96 | 1 | 9 | 0.05 |
| P(Water) | 3.93 | 1 | 9 | 0.05 |
| HAP(Water) | 2.93 | 0.9999 | 9 | 0.05 |

**Supplementary Table S3.** ICC summary

| Parameter | Time (week) | ICC |
| --- | --- | --- |
| Ca(Water) | 0 | 0.929 |
| Ca(Water) | 4 | 0.974 |
| Ca(Water) | 8 | 0.994 |
| P（Water） | 0 | 0.982 |
| P（Water） | 4 | 0.997 |
| P（Water） | 8 | 0.999 |
| HAP（Water） | 0 | 0.970 |
| HAP（Water） | 4 | 0.994 |
| HAP（Water） | 8 | 0.999 |

**Supplementary Table S4.** Between-group comparisons of parameters at week 0 and 4

|  | Experimental group | Control group | *p*_corr |
| --- | --- | --- | --- |
| week 0 |  |  |  |
| Ca(Water)（mg/cm3） | 43.47(43.31,45.49) | 44.04(43.35,45.30) | 1 |
| P(Water)（mg/cm3） | 123.50±4.30 | 122.94±4.54 | 1 |
| HAP(Water)（mg/cm3） | 92.92(92.79,97.30) | 92.82(92.64,96.40) | 1 |
| week 4 |  |  |  |
| Ca(Water)（mg/cm3） | 87.96(86.96,94.15) | 86.89(85.99,87.40) | 0.336 |
| P（Water）（mg/cm3） | 249.37±12.13 | 240.64±3.77 | 0.168 |
| HAP（Water）（mg/cm3） | 191.70±9.21 | 185.02±2.80 | 0.162 |

**Supplementary Table S5.** Spectral CT Parameters at 0, 4, and 8 Weeks

| Parameters | Experimental group | | |  | Control group | | |
| --- | --- | --- | --- | --- | --- | --- | --- |
|  | week 0 | week 4 | week 8 |  | week 0 | week 4 | week 8 |
| Ca(Water)（mg/cm^3^） | 43.47(43.31,45.49)^bc^ | 87.96(86.96,94.15)^ac^ | 123.28±1.91^ab^ |  | 44.04(43.35,45.30)^bc^ | 86.89(85.99,87.40)^ac^ | 113.42±2.96^ab^ |
| P（Water）（mg/cm^3^） | 123.50±4.30^bc^ | 249.37±12.13^ac^ | 341.09±5.28^ab^ |  | 122.94±4.54^bc^ | 240.64±3.77^ac^ | 314.11±8.13^ab^ |
| HAP（Water）（mg/cm^3^） | 92.92(92.79,97.30)^bc^ | 191.70±9.21^ac^ | 261.40(258.80,266.30)^ab^ |  | 92.82(92.64,96.40)^bc^ | 185.02±2.80^ac^ | 240.10(232.70,245.70)^ab^ |

^a^ Compared with week 0, *p*_corr < 0.05; ^b^ Compared with week 4, *p*_corr < 0.05; ^c^ Compared with week 8, *p*_corr < 0.05.

**Supplementary Table S6.** Intra-group comparisons of rates of change (FDR-corrected)

| Parameters | Rate of change_0-4_ | Rate of change_4-8_ | *P*-RDF |
| --- | --- | --- | --- |
| Experimental group |  |  |  |
| Ca(Water) | 1.03±0.08 | 0.37±0.05 | ＜0.001* |
| P（Water） | 1.02±0.08 | 0.37±0.05 | ＜0.001* |
| HAP（Water） | 1.02±0.08 | 0.37±0.05 | ＜0.001* |
| Control group |  |  |  |
| Ca(Water) | 1.00(0.92,1.03) | 0.31±0.02 | 0.004* |
| P（Water） | 1.00(0.91,1.02) | 0.31±0.02 | 0.004* |
| HAP（Water） | 1.00(0.91,1.02) | 0.31(0.26, 0.32) | 0.004* |

*Statistically significant difference
